# Sex-specific associations of serum testosterone with gray matter volume and cerebral blood flow in midlife individuals at risk for Alzheimer’s disease

**DOI:** 10.1371/journal.pone.0317303

**Published:** 2025-01-13

**Authors:** Matilde Nerattini, Schantel Williams, Caroline Andy, Caroline Carlton, Camila Zarate, Camila Boneu, Francesca Fauci, Trisha Ajila, Steven Jett, Michael Battista, Silky Pahlajani, Valentina Berti, Randolph Andrews, Dawn C. Matthews, Jonathan P. Dyke, Roberta Diaz Brinton, Lisa Mosconi

**Affiliations:** 1 Department of Neurology, Weill Cornell Medicine, New York, NY, United States of America; 2 Department of Experimental and Clinical Biomedical Sciences, Nuclear Medicine Unit, University of Florence, Florence, Italy; 3 Department of Population Health Sciences, Weill Cornell Medicine, New York, NY, United States of America; 4 Department of Radiology, Weill Cornell Medicine, New York, NY, United States of America; 5 ADM Diagnostics, Chicago, IL, United States of America; 6 Department of Neurology and Pharmacology, University of Arizona, Tucson, AZ, United States of America; University of Medicine & Dentistry of NJ - New Jersey Medical School, ISRAEL

## Abstract

Testosterone, an essential sex steroid hormone, influences brain health by impacting neurophysiology and neuropathology throughout the lifespan in both genders. However, human research in this area is limited, particularly in women. This study examines the associations between testosterone levels, gray matter volume (GMV) and cerebral blood flow (CBF) in midlife individuals at risk for Alzheimer’s disease (AD), according to sex and menopausal status. A cohort of 294 cognitively normal midlife participants, 83% female, ages 35–65 years, with an AD family history and/or Apolipoprotein E epsilon 4 (APOE-4) genotype, underwent volumetric Magnetic Resonance Imaging (MRI) to measure GMV and MR-Arterial Spin Labeling (ASL) for measurement of CBF. We used voxel-based analysis and volumes of interest to test for associations between testosterone (both total and free testosterone) and brain imaging outcomes, stratified by sex and menopausal status. Higher total and free testosterone levels were associated with larger GMV in men, with peak effects in frontal and temporal regions. Conversely, in women, higher testosterone levels correlated with higher CBF, with peak effects in frontal and limbic regions, subcortical areas and hypothalamus. Among women, associations between testosterone and GMV were observed at the premenopausal and perimenopausal stages, but not postmenopause, whereas associations of testosterone with CBF were significant starting at the perimenopausal stage and were more pronounced among hormone therapy non-users. Results were independent of age, APOE-4 status, midlife health indicators, and sex hormone-binding globulin levels. These findings indicate sex-specific neurophysiological effects of testosterone in AD-vulnerable regions in midlife individuals at risk for AD, with variations observed across sex and menopausal status. This underscores the need for further research focusing on the neuroprotective potential of testosterone in both sexes.

## Introduction

Testosterone, an essential sex steroid hormone, exerts physiological effects either directly or through its aromatization to estradiol (E2) throughout the body and brain [1]. Increasing evidence suggests beneficial effects of testosterone on neurophysiology and neuropathology across the lifespan, potentially influencing the risk of neurological disorders such as Alzheimer’s disease (AD). In the brain, testosterone has documented antioxidant and antiapoptotic properties, modulates excitability and promotes synaptogenesis, exerting broad neurotrophic effects, including cell differentiation and neurogenesis in both male and female animals [2], with some studies indicating sex-differential effects [3,4]. Additionally, preclinical studies have shown that testosterone treatment prevents tau hyperphosphorylation in rats [5] and reduces amyloid beta (Aβ) production in cell cultures [6].

Despite its reputation as a ‘male’ hormone, testosterone is important for both sexes [4]. Women, while having lower absolute levels of circulating testosterone compared to men, maintain higher relative concentrations of testosterone to estradiol throughout their lifespan [7]. Maximum testosterone levels typically occur in the third and fourth decades of life, followed by a gradual decline with age [7]. Additionally, midlife women experience shifts from higher estrogen levels to altered androgen-to-estrogen ratios after menopause, especially following oophorectomy (surgical menopause) [7]. Neuroimaging studies have identified the menopause transition as a female-specific risk factor for AD [8–11], and testosterone deprivation as an AD risk factor for men [12,13].

However, our understanding of testosterone’s role in human neurophysiology is limited, especially in women, as most research has focused on men. In elderly men, higher testosterone levels have been associated with better cognitive performance across various domains [14,15] and reduced risk of cognitive impairment and AD [12,13]. Some brain imaging studies also indicate positive associations between testosterone levels and brain health indicators including gray matter volume (GMV) [16,17], cerebral blood flow (CBF) [18], and glucose metabolism [19] in old age, whereas such associations were less evident or absent in midlife men [20–22]. On the other hand, studies examining associations of testosterone with Aβ load in elderly men, both with and without cognitive impairment, have reported inconsistent results. One study found associations between free testosterone and hippocampal volume but not with Aβ load, which were limited to patients with mild cognitive impairment (MCI) or AD [23]. Another study reported associations with Aβ burden in MCI patients but found no associations among cognitively normal elderly or AD patients [24].

Conversely, the few imaging studies investigating testosterone’s impact on GMV in women, including midlife [25,26] and older participants [17,27], have reported no significant effects. One study found an inverse relationship between free testosterone and cerebral Aβ positivity, though no effects on hippocampal volume, across elderly women with and without MCI or AD [23]. Studies of younger women in their 30’s have yielded mixed results, with either null [28] or positive associations with hippocampal volume [20], and negative associations with regional CBF [22]. These studies did not specifically focus on women at risk for AD, who may exhibit different biomarker profiles. Overall, there is a notable gap in imaging studies exploring the associations of testosterone levels with brain biomarkers in midlife individuals at risk for AD, particularly women, and how these associations may differ by menopausal status.

Herein, we used volumetric MRI and Arterial Spin Labeling (ASL) to examine sex-specific associations of serum testosterone (both total and free testosterone) with regional GMV and CBF in cognitively normal midlife women and men at risk for late-onset AD (family history and/or Apolipoprotein E epsilon 4, APOE-4, genotype). We also examined whether these associations vary according to menopausal status and hormone therapy usage.

## Methods

### Participants and data

This is a natural history, non-interventional study of cognitively normal men and women ages 35–65 years, with a family history of late-onset AD and/or Apolipoprotein E epsilon 4 (APOE-4) genotype, both major genetic risk factors for late-onset AD [29]. Among participants enrolled at the Weill Cornell Medicine (WCM) Alzheimer’s Prevention Program, subjects who completed clinical and cognitive examinations, laboratory tests, endocrine status assessments, and brain imaging between January 1, 2018, and June 30, 2023, were included in the current study.

Our inclusion and exclusion criteria have been previously described [30–34]. Briefly, all participants had Montreal Cognitive Assessment (MoCA) score >26 and normal cognitive test performance by age and education. Exclusion criteria were medical conditions that may affect brain structure or function (e.g., stroke, any neurodegenerative diseases, and major psychiatric disorders such as major depressive disorder, schizophrenia, or bipolar disorder). All participants received clinical, laboratory, cognitive exams, and MRI scans (volumetric MRI and Arterial Spin Labeling, ASL). Additionally, clinical MRI scans were inspected by an expert neuroradiologist to rule out evidence of incidental findings including hydrocephalus, demyelinating disorders, generalized atrophy, intracranial mass, infarcts and lacunes.

APOE-4 genotype was determined using standard qPCR procedures [30–35]. Participants carrying one or two copies of the APOE-4 allele were grouped together as APOE-4 carriers and compared to non-carriers. A family history of late-onset AD was elicited using standardized questionnaires [30–35].

The patients’ sex was determined by self-report. Determination of menopausal status was based on the Stages of Reproductive Aging Workshop (STRAW) criteria [36] with hormone assessments as supportive criteria [34]. Participants were classified as premenopausal (regular cycler), perimenopausal (irregular cyclers with interval of amenorrhea ≥60 days or ≥2 skipped cycles) and postmenopausal (no cycle for ≥12 months) [34]. Information on hysterectomy/ oophorectomy status and hormone therapy (HT) usage, including menopausal hormone therapy (MHT) and oral contraceptives (OCP), was obtained by the study physician through review of medical history.

### Standard protocol approvals, registrations, and patient consents

All experimental protocols were approved by the WCM Institutional Review Board. Written informed consent was obtained from all participants. Data were analyzed anonymously, and all experiments were performed in accordance with the relevant guidelines and regulations.

### Cognitive measures

Participants underwent a cognitive testing battery, assessing memory, language, fluency, and executive function [30–33].

#### Memory

We assessed encoding and retention using the Rey Auditory Verbal Learning Test (RAVLT) [37]. Participants were presented a list of 15 words over 5 trials. Recall was assessed after a short and a 15-minute delay. We used the Logical Memory subtest of the Wechsler Memory Scale to assess narrative episodic memory [38]. A short story was orally presented, and the participant was asked to recall the story verbatim immediately after hearing it (immediate recall). Approximately 15 or 20 min later, the participant was asked to recall the story again (delayed recall) to assess the retention of verbal information over time. We used total scores for both subtests for analysis.

#### Executive function

We used the Trail Making Test (TMT) to assess executive function [39]. It involves connecting 13 dots numbered 1–13 and 12 letters A–L, alternating numbers and letters, across a page in increasing order. The TMT Part B measures a person’s ability to shift between cognitive sets, with higher scores reflecting poorer performance.

#### Verbal fluency

We assessed verbal fluency using FAS and animal tests [40]. Participants were asked to name as many words as possible beginning with a given letter (e.g., F, A, S), and fitting a specified semantic category (animals), within a 60-second time limit. We used the total number of words generated for each task.

#### Language

We used the Boston naming test (BNT) [41] to gauge language function, specifically a person’s ability to retrieve words in response to visual confrontation. It consists of 60 line-drawn objects of decreasing familiarity, presented in order of difficulty from "easiest" (e.g., "tree") to "most difficult" (e.g., "abacus"). Participants have 20 seconds to name each item. A total score is then calculated.

### Hormonal panel

All participants received a blood draw by venipuncture after an overnight fast. Samples were shipped overnight to CLIA-certified Boston Heart Diagnostics [Framingham, MA] and analyzed on a Roche Cobas e801 analytical unit for immunoassay tests using Electrochemiluminescence technology (ECL) [Roche Diagnostics; Basel, Switzerland]. Total testosterone (totT) and sex hormone-binding globulin (SHBG) were assessed trough electrochemiluminescence immunoassay “ECLIA”, in accordance with the Clinical and Laboratory Standards Institute EP17-A2 requirements. For totT, the measuring range was 0.087 to 52.0 nmol/L (2.50-1500 ng/dL), with within run precision of <1.7% and between run precision of <1.9% at 886 ng/dL. For SHBG, the measuring range was 0.8 to 200 nmol/L, with within run precision of <1.4% and between run precision of < 3.1% at 47.7 nmol/L. Free testosterone (freeT) levels were calculated by means of individual concentrations for totT, SHBG, and albumin, and via the association constant of albumin to testosterone [42].

### Image acquisition and analysis

Participants received brain MRI scans on a 3.0 Tesla G.E. MR 750 Discovery scanner (General Electric, Waukesha, WI) equipped with a 32-channel head coil, following standardized procedures [30–34], including a 3D volumetric T1-weighted MRI scan [Brain Volume Imaging (BRAVO); 1x1x1 mm resolution, 8.2 ms repetition time (TR), 3.2 ms echo time (TE), 12° flip angle, 25.6 cm field of view (FOV), 256x256 matrix with ARC acceleration], and an Arterial Spin Labeling (ASL) scan [pseudo-continuous (pc) technique with 4851 ms TR, 10.6 ms TE, 4 averages, 128x128 reconstruction matrix, acquisition resolution of 3.64x3.64x3.8mm typically performed on the same day. pcASL image data were reconstructed in the Cartesian plane at 1.875mm x 1.875mm, with a slice thickness of 3.8mm. ASL scans from ten participants were excluded due to truncation artifact after warping (n = 8) or suboptimal preprocessing quality (n = 2).

Image analysis was performed using a fully automated image processing pipeline [30–34]. Volumetric MRI, and ASL scans were realigned and coregistered to each other using the Normalized Mutual Information cost function of Statistical Parametric Mapping (SPM12) [43] implemented in Matlab R2018a (MathWorks; Natick, MA). The MRI scans were spatially normalized to the template tissue probabilistic map (TPM) image in SPM12, conforming to the Montreal Neurological Institute (MNI) space, and processed for voxel-based morphometry (VBM) [44] including image segmentation, Jacobian modulation, high-dimensional warping (DARTEL) of the segments, and application of an 8mm full-width at half maximum (FWHM) smoothing kernel [44]. Gray matter (GM) segments were retained for statistical analysis. Secondly, the MRI-coregistered ASL scans were spatially normalized to the TPM image using the corresponding MRI subject-specific transformation matrices, using the MRI as the anchor, and smoothed using an 8-mm FWHM filter. FreeSurfer 7.2 [45] was utilized to calculate total intracranial volume (TIV) for normalization purposes [44].

### Covariates

Cognitive analyses were adjusted by age and education (years). Imaging analyses were adjusted by (Model 1) age and modality-specific confounders (TIV for GMV, global mean for CBF); and additionally, by (Model 2) APOE-4 status (carrier vs. non-carrier) and midlife health indicators, including smoking (current vs. past vs. never smoker), hypercholesterolemia (normal vs. borderline cholesterol 200–240 mg/dL vs. hypercholesterolemia > 240 mg/dL), waist to hip ratio (abnormal if > 0.90, male and > 0.83, female), medically controlled Type 2 diabetes (positive vs. negative), and medically controlled hypertension (positive vs. negative). As totT includes both active (unbound) and inactive (bound) testosterone, SHBG (nmol/L) was included as an additional covariate in analysis of totT to enhance the biological plausibility of the results.

### Statistical analysis

Analyses were performed in R v.4.2.0 and SPM12.

Clinical measures were examined using general linear models or chi-squared tests as appropriate. Cohort characteristics are described using mean (standard deviations, SD) and n, percentage (%), stratified by group. As the hormone measures did not follow a Gaussian distribution, we used the automated bestNormalize R package to identify the optimal normalization transformation for each measure. In men, the standardized sqrt (x + a) transformation was selected for both totT and freeT data as it provided the best goodness of fit. In women, the standardized Yeo-Johnson transformation was selected for freeT levels, while orderNorm transformation was applied to totT measures. Following the transformations, all variables passed the Shapiro Wilks test for normality and their histograms displayed normal distributions.

#### Testosterone associations with brain biomarkers

We used multivariate linear regressions with post-hoc t-contrasts, as implemented in SPM12, to test for voxel-based associations between exposures and biomarker outcomes (GMV and CBF), adjusting by covariates. TotT was examined as the main exposure. Analyses were performed separately for men and for women. For women, analyses were performed in the entire cohort and after excluding hormone therapy (HT) users. These procedures were then used to test for associations between totT and imaging outcomes in women at different menopausal stages. This analysis was restricted to HT non-users, as HT may impact brain biomarker levels [7].

For all analyses, statistical maps were obtained at P<0.05, cluster-level corrected for Family-Wise Type Error (FWE) within a binary masking image consisting of *a priori* defined regions with known vulnerability to AD (AD_MASK_), including frontal, parietal, temporal cortex, cingulate gyrus and precuneus, thalamus and medial temporal lobes [46]. The AD_MASK_ was set as an explicit (inclusive) mask to conservatively restrict analysis to regions within the mask [43]. Cluster extent was set at >20 voxels (e.g., twice or greater the scans’ effective spatial resolution), to further reduce the likelihood of Type I errors [47]. For exploratory purposes, results were also examined outside of the masking image, at an uncorrected threshold of P<0.001.

FreeT was examined as a secondary exposure. As such, analyses were restricted to the voxels with statistically significant associations between totT and imaging outcomes (GMV or CBF), thereby limiting excessive hypothesis testing. This was accomplished as detailed in **S1 Fig** and described in [48]: (a) we used linear regressions to test for voxel-wise associations between totT and imaging biomarkers across all participants; (b) Regional clusters showing totT effects on outcome measures were saved as a binary map (totT_MASK_); (c) We used linear regressions to test for voxel-wise associations between freeT and imaging biomarkers across all participants within the totT_MASK_. This was done by setting totT_MASK_ as an explicit mask, e.g. including only voxels residing within the mask [32].

Anatomical location of regions reaching significance was described using MNI coordinates and Automated Anatomical Labelling atlas 3 (AAL3) regions. Biomarker measures were extracted from peak clusters as volumes-of-interest (VOI) using MarsBar 0.45 [https://marsbar-toolbox.github.io/download.html] for further analysis.

### Sensitivity analysis

#### Hypothalamus

Given evidence that voxel-based analysis may underestimate effects in small brain regions [49], we also tested for associations between exposures and outcomes in the hypothalamus–a structure with well-established neuroendocrine functions–using the VOI approach. Fitting of the hypothalamus VOI was confirmed on the coregistered anatomical MRI by two expert raters (MN, VB). Regression models were trained to test for associations between exposures (totT or freeT) and hypothalamic volume and CBF, adjusting for covariates, at *P*<0.05.

#### Effects of Hormone Therapy (HT)

To investigate regional differences in the covariate-adjusted correlation of totT and CBF outcomes by HT status (users vs. non-users), partial correlations were calculated and displayed in a heat map. Then, to determine whether HT significantly modified the relationship between totT and CBF, a Multivariate Analysis of Covariance (MANCOVA) test was performed. Covariates included the full covariate panel, as well as totT, HT status, and their interaction. Scatterplots of totT and CBF stratified by HT status were generated for those brain regions showing significant interaction effects. This analysis was restricted to perimenopausal and postmenopausal women and was limited to CBF measures which demonstrated significant main effects of totT.

#### Associations of testosterone and cognition

Cognitive measures were standardized prior to analysis. To determine whether testosterone was significantly correlated with cognition, a Multivariate Analysis of Covariance (MANCOVA) test was performed, testing totT measure against the multivariate cognition outcome, controlling for covariates, at P<0.05. Due to the smaller sample size for men, individual partial correlation p-values were calculated descriptively for each brain region, in lieu of performing a MANCOVA test. Additionally, we used regression analyses to test for associations between regional biomarkers showing significant associations with totT and cognitive test scores, to explore possible mediation effects.

## Results

### Participants

We enrolled 310 participants for this study. Of these, 16 were excluded due to incidental findings on Magnetic Resonance Imaging (MRI) (n = 3), scan artifacts (n = 2), or incomplete hormonal test results (n = 11). The remaining 294 participants were examined in this study, including 51 men, 62 premenopausal, 87 perimenopausal and 94 postmenopausal women.

Participant characteristics are shown in **Table 1**. The study cohort was in good general health, with only a small percentage of individuals with medically controlled diabetes (5%), hypercholesterolemia (10%), history of mild depression (30%), and/or history of hypertension (10%). All participants carried risk factors for late-onset AD, including 98% with a family history of AD and 46% APOE-4 carriers. Among postmenopausal women, 9% (n = 22) had a history of hysterectomy or oophorectomy. Thirty-two percent of women reported taking HT and none reported testosterone supplementation. None of the men reported taking hormones.

**Table 1:** Participants’ characteristics.

	Women	Men
N	243	51
Age, years	51 (6)	50 (8)
Education, years	17 (2)	18 (2)
Race, % white	81	73
MoCA score, unitless	28 (1)	29 (1)
History of mild depression, % positive	31	24
APOE-4 carrier status, % positive	44	57
AD family history, % positive	98	98
Menopause status, % premenopause / perimenopause / postmenopause	26 / 35/ 39	n.a.
Hormone therapy, % current users	32	n.a.
Hysterectomy / oophorectomy status, % positive	9	n.a.
Hormone levels		
Total Testosterone (ng/dL)	13(10)*	514 (152)
Free Testosterone (pg/mL)	2(2)*	77 (21)
SHBG (nmol/L)	90(42)*	52 (23)
Midlife health risks		
Waist to hip ratios, unitless	0.83 (0.10)*	0.93 (0.06)
Hypercholesterolemia, % borderline / positive	43 / 11*	22 / 3.9
Type 2 diabetes, % positive	3.4	2.0
Smoking status, % current / past	1.3 / 23	2.0 / 18
Hypertension, % positive	7*	21
Cognitive testing, unitless		
Logical memory, delayed recall	13 (4)	14 (4)
Logical memory, immediate recall	15 (4)	15 (4)
RAVLT, delayed recall	10 (3)	10 (3)
RAVLT, recognition	14 (2)	14 (2)
FAS	49 (12)	50 (12)
Animal naming	25 (6)	24 (5)
Boston naming test	14 (1)	14 (1)
Trail Making Test B	54 (22)	66 (92)

Values are mean (standard deviations) unless otherwise specified.

*Different from men, P<0.05.

There were no sex differences in demographic, clinical or cognitive measures except for a higher presence of central obesity and hypertension in men (*P* = 0.015), and a higher presence of hypercholesterolemia in women (*P*<0.001). As expected, men showed higher totT and freeT levels compared to women (*P*<0.001), while women showed higher serum SHBG levels (*P*<0.001).

### Sex-specific associations between testosterone levels and gray matter volume

**Fig 1:**
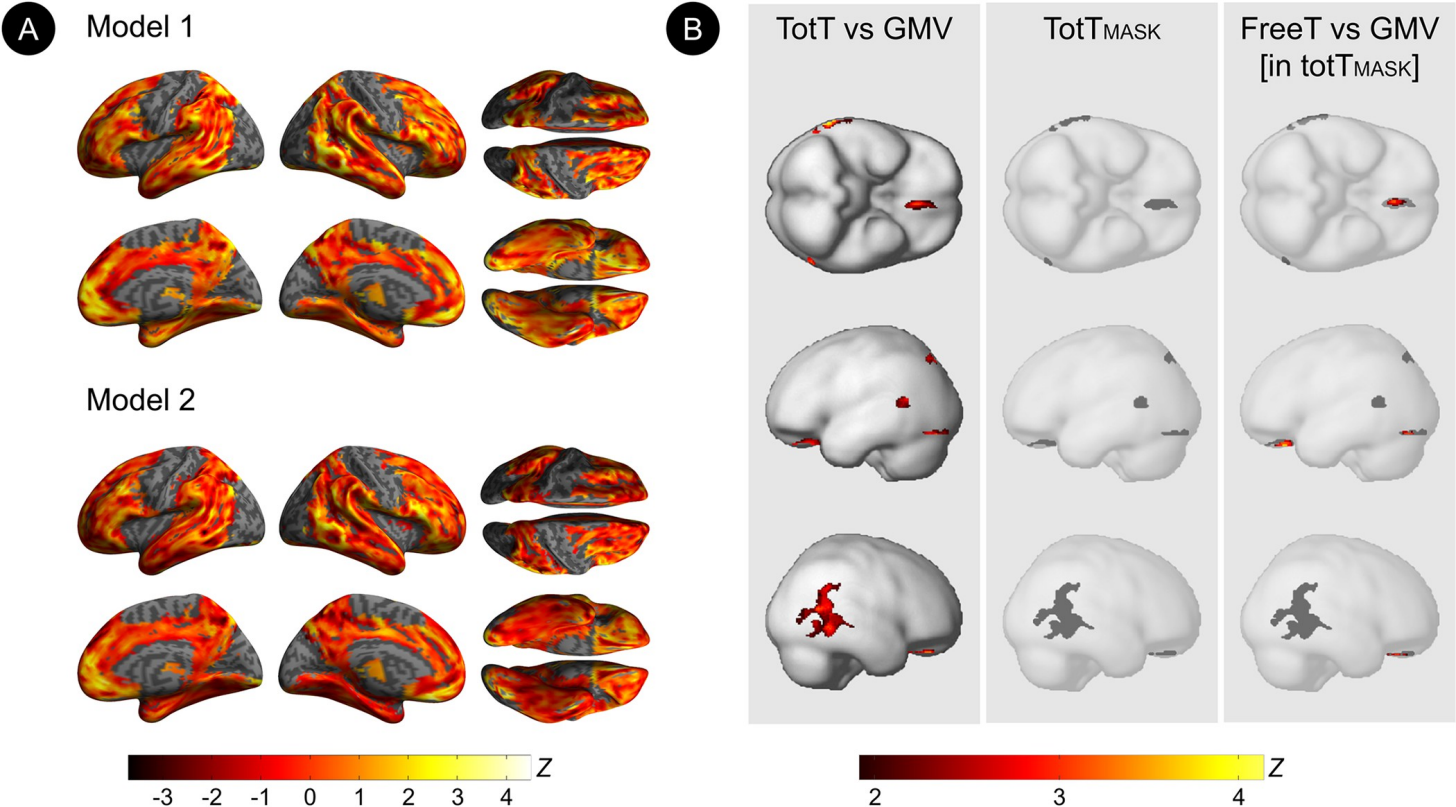
Associations between serum testosterone and gray matter volume in AD-regions among men. (A) Surface maps of voxel-wise associations of total testosterone (totT) and MRI gray matter volume (GMV) among men, adjusting by total intracranial volume and age (model 1), APOE-4 status, midlife health indicators and sex hormone-binding globulin (model 2). Surface maps are generated at P<0.05, cluster-level corrected for Family-Wise Type Error (FWE) within the prespecified search volume of AD-vulnerable regions, and displayed on the lateral, superior and inferior views of an inflated, rendered MR image. (B) Results from subtraction analysis are displayed on the inferior and lateral views of a volume-rendered MR template image:
*Left*. Statistical parametric maps (SPMs) displaying positive voxel-wise associations between totT and GMV in parieto-temporal and orbitofrontal regions, restricted to the peaks of statistical significance.*Middle*. Clusters with statistically significant associations of totT with GMV are saved as a binary masking image (totT_MASK_).*Right*. SPMs displaying associations of free testosterone (freeT) with GMV within the totT_MASK_ image.Results are displayed using color-coded scales with corresponding Z score values. N = 51 men were included in this study.

Among men, totT was positively associated with gray matter volume (GMV) in middle temporal gyrus, bilaterally; orbitofrontal cortex and fusiform gyrus of the left hemisphere; and right superior temporal gyrus (multivariable adjusted *P*_*FWE*_ *≤*0.023**; Table 2, Fig 1A**). FreeT was positively associated with GMV in orbitofrontal cortex and fusiform gyrus of the left hemisphere (multivariable adjusted *P*_*FWE*_ *≤*0.005**; Table 2, Fig 1B).** Adjusting for the same confounders, there were no brain regions exhibiting negative associations of totT or freeT with GMV (**Table 2**).

**Table 2:** Associations between testosterone and regional gray matter volume among men.

Cluster extent	Coordinates x, y, z	Z	P_FWE_ cluster*	P voxel	Anatomical Region
**Total Testosterone**					
***Positive associations***					
65	-6 33–27	4.02	0.010	<0.001	Orbitofrontal cortex, left
105	70–46–2	3.64	0.005	<0.001	Middle temporal gyrus, right
30	-64–54 12	3.57	0.021	<0.001	Middle temporal gyrus, left
70	64–51 18	3.47	0.009	<0.001	Superior temporal gyrus, right
25	-39–82–18	3.34	0.023	<0.001	Fusiform gyrus, left
***Negative associations***					
n.s.					
**Free testosterone**					
***Positive associations***					
43	-6 33–26	4.09	0.003	<0.001	Orbitofrontal cortex, left
22	-40–75–18	3.70	0.005	<0.001	Fusiform gyrus, left
***Negative associations***					
n.s.					

*P<0.*05* cluster-level corrected for Family-Type Wise Error (FWE) within the search volume. Analyses were adjusted by age, total intracranial volume, APOE-4 status, midlife health indicators and SHBG.

Among women, there were no brain regions exhibiting significant positive or negative associations of totT or freeT with GMV, in the entire cohort or among HT non-users.

### Sex-specific associations between testosterone levels and cerebral blood flow

Among men, there were no significant positive or negative associations between testosterone levels and regional CBF.

**Fig 2:**
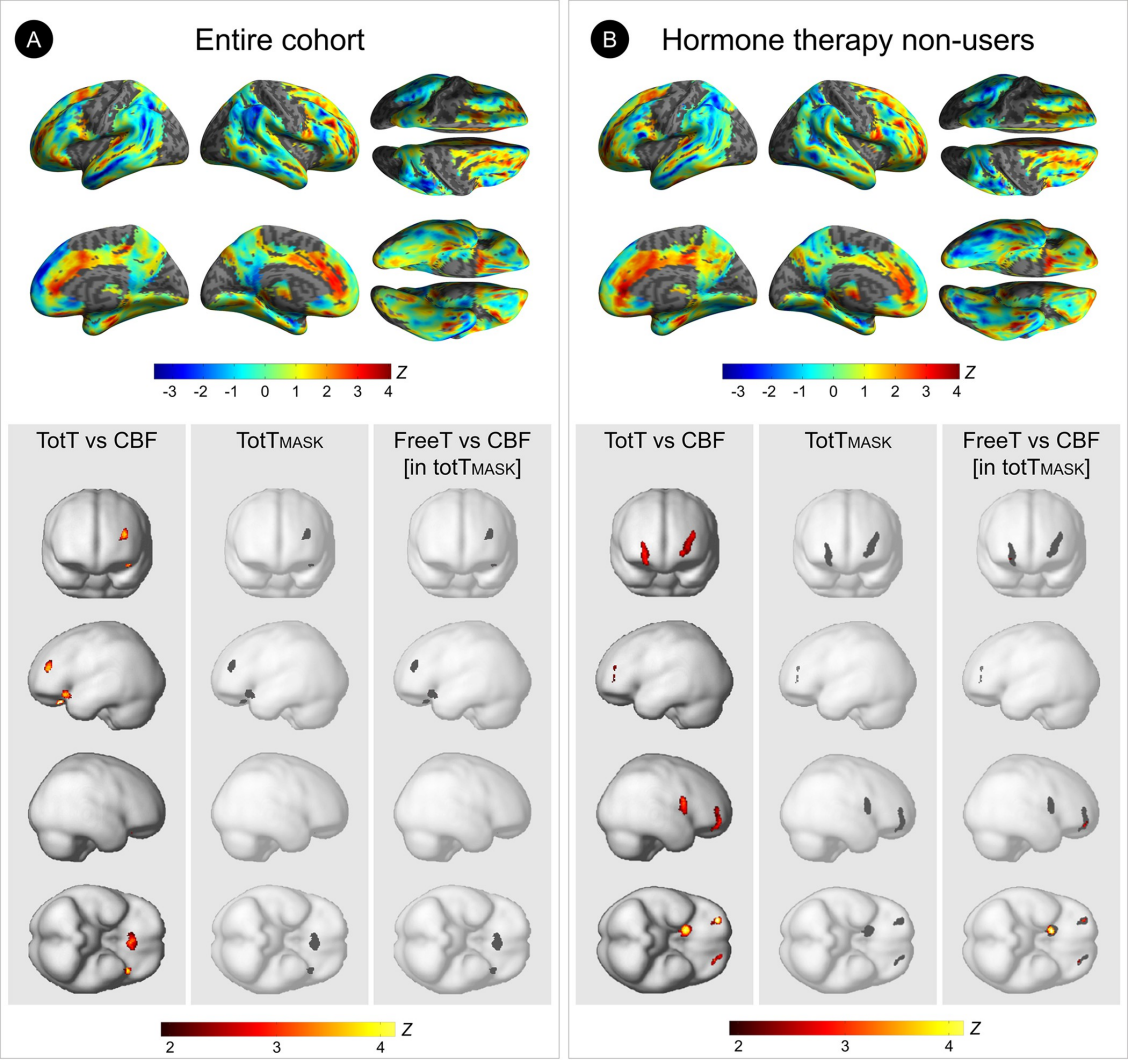
Associations between serum testosterone and cerebral blood flow in AD-regions among women. Results for (A) the entire cohort and (B) hormone therapy (HT) non-users. Each panel displays two sets of results: *Top*. Surface maps of voxel-wise associations of total testosterone (totT) and cerebral blood flow (CBF), adjusting by age and global CBF. Surface maps are generated at P<0.05, cluster-level corrected for Family-Wise Type Error (FWE) within the prespecified search volume of AD-vulnerable regions, and displayed on the lateral, superior and inferior views of an inflated, rendered MR image. *Bottom*. Results from subtraction analysis are displayed on the inferior and lateral views of a volume-rendered MR template image:
Left: Statistical parametric maps (SPMs) displaying voxel-wise associations between totT and CBF.Middle: Voxels with statistically significant totT-CBF associations are saved as a binary masking image (totT_MASK_).Right: SPMs displaying associations of free testosterone (freeT) with CBF within the totT_MASK_. Results are displayed using color-coded scales with corresponding Z values. N = 243 women were included in this study, including 79 HT users.

Among women, in the entire cohort, totT levels were positively associated with CBF in cingulate cortex, hippocampus, thalamus, middle, inferior and orbital frontal cortices, and superior temporal gyrus of the left hemisphere (multivariable adjusted *P*_*FWE*_ *≤*0.024**; Table 3, Fig 2A**). Restricting analysis to HT non-users resulted in more widespread positive associations of totT with CBF in bilateral superior frontal gyri; hippocampus, thalamus, and middle frontal gyrus of the left hemisphere; and putamen, anterior and middle cingulate cortices, and orbitofrontal cortex of the right hemisphere (multivariable adjusted *P*_*FWE*_ *≤*0.050**; Table 3, Fig 2B)**.

**Table 3:** Associations between total testosterone and regional cerebral blood flow in AD-regions among women.

Cluster extent	Coordinates x, y, z	Z	P_FWE_ cluster*	P voxel	Anatomical Region
**Entire cohort**					
***Positive associations***					
96	-6 21 38	3.76	0.010	<0.001	Midcingulate cortex, left
125	-18–34–4	3.74	0.008	<0.001	Hippocampus, left
	-12–33 3	3.43		<0.001	Thalamus, left
53	-34 45 21	3.60	0.016	<0.001	Middle frontal gyrus, left
37	-40 24–15	3.52	0.020	<0.001	Orbitofrontal cortex, left
26	-42–28 9	3.31	0.024	<0.001	Superior temporal gyrus, left
25	-4 27–24	3.25	0.024	<0.001	Inferior frontal gyrus, left
***Negative associations***					
467	54 34 16	4.22	0.01	<0.001	Inferior frontal gyrus, right
	51 46 9	3.46		<0.001	Middle frontal gyrus, right
	56 44 0	3.23		<0.001	Inferior frontal gyrus, right
53	-16 24 10	3.46	0.030	<0.001	Caudate, left
**HT non-users**					
***Positive associations***					
231	-21–36–4	4.55	0.007	<0.001	Hippocampus, left
	-15–33 0	3.97		<0.001	Thalamus, left
373	14 12–9	3.90	0.003	<0.001	Putamen, right
92	-3–16 0	3.81	0.023	<0.001	Thalamus, left
95	6 40–2	3.41	0.023	<0.001	Anterior cingulate cortex, right
28	4 6 42	3.38	0.050	<0.001	Midcingulate, right
34	27 52–15	3.27	0.046	<0.001	Orbitofrontal cortex, right
	27 57–8	3.17		<0.001	Superior frontal gyrus, right
31	-27 48 0	3.26	0.048	<0.001	Superior frontal gyrus, left
	-28 48 9	3.16		<0.001	Middle frontal gyrus, left
***Negative associations***					
304	54 36 16	3.68	0.002	<0.001	Inferior frontal gyrus, right
	52 45 4	3.37		<0.001	Middle frontal gyrus, right
27	-63–8–9	3.51	0.025	<0.001	Middle temporal gyrus, left
38	-63–9–27	3.39	0.021	<0.001	Middle temporal gyrus, left

*P<0.*05* cluster-level corrected for Family-Type Wise Error (FWE) within the search volume Analyses were adjusted by age, global mean CBF, APOE-4 status, midlife health indicators and SHBG.

In both analyses, negative associations of totT and CBF were observed in middle and inferior frontal gyri of the right hemisphere (multivariable adjusted *P*_*FWE*_ *≤*0.030**; Table 3, Fig 2A and 2B).**

There were no regions exhibiting associations of freeT with CBF in the entire cohort, whereas among HT non-users, freeT levels were positively associated with CBF in hippocampus of the left hemisphere, and orbitofrontal cortex and putamen of the right hemisphere (multivariable adjusted *P*_*FWE*_ *≤*0.018**; S1 Table, Fig 2B**).

### Effects of menopausal status on the associations of testosterone with brain biomarkers

**Fig 3:**
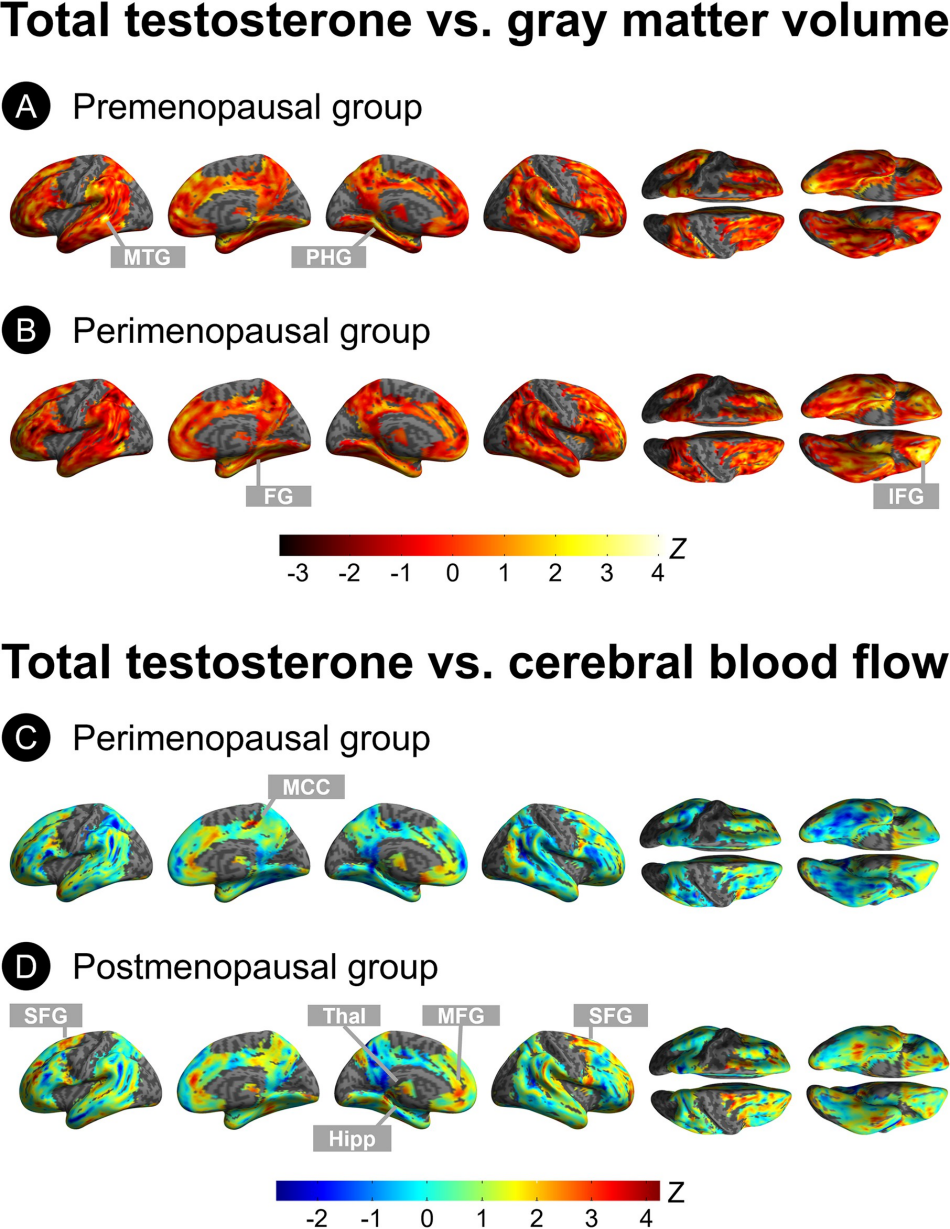
Effects of menopausal status on the associations of total testosterone with gray matter volume and blood flow. Surface maps displaying voxel-wise associations of total testosterone with (A-B) MRI gray matter volume and (C-D) cerebral blood flow by menopause status, adjusted by modality-specific confounders, age, APOE-4 status, midlife health risks and sex hormone-binding globulin (SHBG). Results are generated at P<0.05, cluster-level corrected for Family-Wise Type Error (FWE) within the prespecified search volume of AD-vulnerable regions, and displayed on the lateral, superior and inferior views of an inflated, rendered MR image. Only significant results are shown in the figure. This analysis was restricted to the 164 women HT non-users. Abbreviations: FG: Fusiform gyrus; Hipp: Hippocampal cortex; IFG: Inferior frontal gyrus; MCC: Midcingulate cortex; MFG: Middle frontal gyrus; MTG, middle temporal gyrus; PHG, parahippocampal gyrus; SFG: Superior frontal gyrus; Thal: Thalamus.

***Gray matter volume*.** Results are shown in **Table 4** and **Fig 3.** The premenopausal group exhibited positive associations between totT and GMV in middle temporal cortex and parahippocampal gyrus of the left hemisphere (multivariable adjusted *P*_*FWE*_ = 0.004, **Fig 3A**). The perimenopausal group exhibited positive associations of totT with GMV in right fusiform gyrus, and middle and inferior frontal cortices of the left hemisphere (multivariable adjusted *P*_*FWE*_ ≤0.007, **Fig 3B**). The postmenopausal group exhibited no associations of totT with GMV after multivariable adjustment. There were no negative associations between totT and GMV in any group.

**Table 4:** Associations between total testosterone and gray matter volume by menopause status among HT non-users.

Cluster extent	Coordinates x, y, z	Z	P_FWE_ cluster*	P voxel	Anatomical Region
**Premenopausal group**					
***Positive associations***					
27	-56–40–3	3.74	0.004	<0.001	Middle temporal gyrus, left
29	-14–39–6	3.50	0.004	<0.001	Parahippocampal gyrus, left
***Negative associations***					
n.s.					
**Perimenopausal group**					
***Positive associations***					
41	34–9–36	3.84	0.007	<0.001	Fusiform gyrus, right
60	-26 46–12	3.38	0.005	<0.001	Middle frontal gyrus, left
	-32 34–14	3.33		<0.001	Inferior frontal gyrus, left
**Negative associations**					
n.s.					
**Postmenopausal group**					
***Positive associations***					
n.s.					
***Negative associations***					
n.s.					

*P<0.*05* cluster-level corrected for Family-Type Wise Error (FWE) within the search volume. Analyses were adjusted by age, total intracranial volume, APOE-4 status, midlife health indicators, and SHBG.

***Cerebral blood flow***. Results are shown in **Table 5** and **Fig 3**. In the premenopausal group, there were no positive associations between totT and CBF, while negative associations were found in bilateral middle temporal gyrus and superior frontal gyrus of the right hemisphere (multivariable adjusted *P*_*FWE*_ *≤*0.017). In the perimenopausal group, totT was positively associated with CBF in middle cingulate cortex of the right hemisphere (multivariable adjusted *P*_*FWE*_ *=* 0.005, **Fig 3C**). In the postmenopausal group, totT levels exhibited positive associations with CBF in superior frontal cortex, bilaterally; and middle frontal gyrus, hippocampus, and thalamus of the left hemisphere (multivariable adjusted *P*_*FWE*_ *≤*0.031, **Fig 3D**). There were no negative associations of totT with CBF in either perimenopausal or postmenopausal groups.

**Table 5:** Associations between total testosterone and cerebral blood flow by menopause status among HT non-users.

Cluster extent	Coordinates x, y, z	Z	P_FWE_ cluster*	P voxel	Anatomical Region
**Premenopausal group**					
***Positive associations***					
n.s.					
***Negative associations***					
256	52–48 4	3.99	0.001	<0.001	Middle temporal gyrus, right
25	12 44 48	3.64	0.017	<0.001	Superior frontal gyrus, right
27	-50 0–22	3.44	0.017	<0.001	Middle temporal gyrus, left
26	-68–27–3	3.21	0.017	<0.001	Middle temporal gyrus, left
**Perimenopausal group**					
***Positive associations***					
75	18–32 44	3.86	0.005	<0.001	Midcingulate, right
***Negative associations***					
n.s.					
**Postmenopausal group**					
***Positive associations***					
85	-38 45 22	3.88	0.012	<0.001	Middle frontal gyrus, left
66	-20–38–3	3.69	0.016	<0.001	Hippocampus, left
	-14–33 0	3.21		<0.001	Thalamus, left
23	18 15 62	3.51	0.030	<0.001	Superior frontal gyrus, right
21	-15–10 72	3.38	0.031	<0.001	Superior frontal gyrus, left
	-12–3 72	3.29		<0.001	Superior frontal gyrus, left
56	21–8 58	3.32	0.018	<0.001	Superior frontal gyrus, right
36	-21 6 58	3.25	0.024	<0.001	Superior frontal gyrus, left
	-24 15 57	3.22		<0.001	Superior frontal gyrus, left
***Negative associations***					
n.s.					

*P<0.05 cluster-level corrected for Family-Type Wise Error (FWE) within the search volume. Analyses were adjusted by age, global mean CBF, ***APOE***-4 status, midlife health indicators and SHBG.

### Sensitivity analysis

#### Hypothalamus

No significant associations were found between totT or freeT and hypothalamic GMV or CBF in men. No significant associations were observed between totT or freeT and hypothalamic GMV in women, before or after restricting to HT non-users, or by menopausal status. A positive association of totT with CBF was observed (r = 0.167, *P* = 0.023), which was marginally significant after restricting analysis to HT non-users (r = 0.161, *P* = 0.066). On post-hoc analysis, this association was driven by the perimenopausal group (r = 0.317, *P* = 0.011), whereas no significant associations were found in the pre- and postmenopausal groups (**S2 Fig**). No significant associations were found between freeT and hypothalamic CBF. There were no differential effects of HT use on hypothalamic measures (**Table 6**).

**Table 6:** Effects of hormone therapy on the associations of total testosterone with cerebral blood flow among women.

	HT non-users		HT users		TotT by HT status interaction	
	Coeff.	P	Coeff.	P	Coeff.	P
Anterior cingulate cortex	0.372	**<0.001**	-0.035	0.835	-2.974	**0.047**
Cingulate cortex	0.413	**<0.001**	-0.030	0.859	-3.292	**0.017**
Hippocampus	0.457	**<0.001**	0.0284	0.866	-2.198	**0.049**
Putamen	0.429	**<0.001**	-0.098	0.558	-3.044	**0.006**
Superior frontal gyrus, left	0.342	**<0.001**	-0.316	0.053	-5.626	**0.005**
Superior frontal gyrus, right	0.328	**0.001**	-0.372	**0.021**	-6.233	**0.001**
Thalamus	0.385	**<0.001**	0.192	0.249	-3.087	**0.033**
Hypothalamus	0.200	0.056	0.364	**0.025**	-0.180	0.795

Multivariable adjusted coefficients from regression models testing for associations of total testosterone (totT) and cerebral blood flow (CBF) in select regions are displayed by hormone therapy (HT) status (users vs. non-users) and for the interaction terms, with corresponding P values. Significant P values are in bold.

#### Effects of hormone therapy

**Fig 4:**
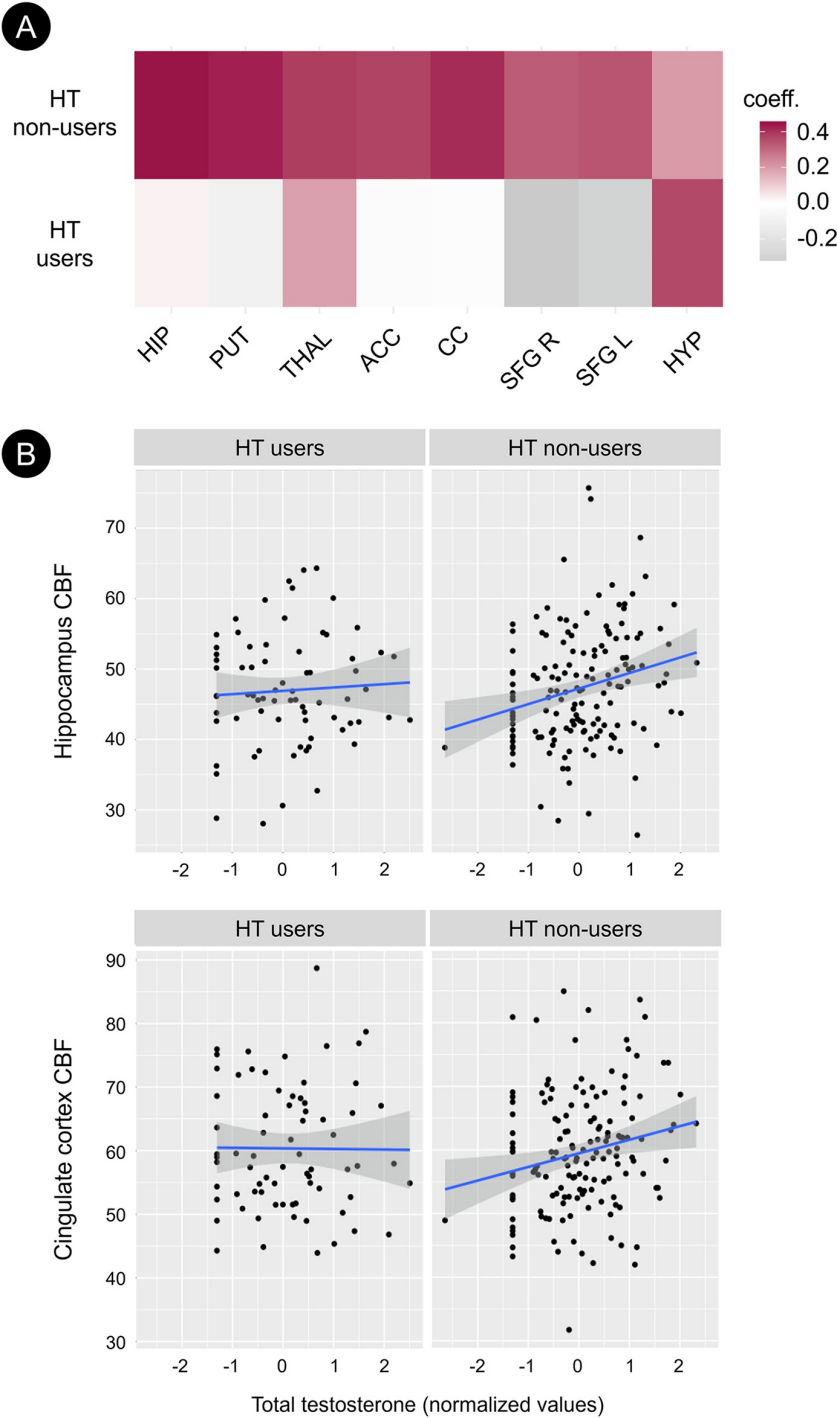
Effects of hormone therapy on the associations of total testosterone with cerebral blood flow among women. (A) Heat maps showing covariate adjusted partial correlation of total testosterone (totT) and cerebral blood flow (CBF) in the hormone therapy (HT) users and HT non-users cohorts. (B) Scatterplots of totT and hippocampal and cingulate cortex CBF stratified by HT status. HT non-users (right exhibit a significant positive association of totT with CBF, whereas HT users (left) do not. Abbreviations: ACC, anterior cingulate cortex, CC: Cingulate cortex, HIP: Hippocampus, HYP: Hypothalamus, L: Left, PUT: Putamen, R: Right, SFG: Superior frontal gyrus, THAL: Thalamus.

Results are shown in **Table 6** and **Fig 4**. This analysis was restricted to biomarker measures showing significant associations with totT (see Methods). From a MANCOVA test comparing a multivariate CBF outcome across all brain regions and adjusting for covariates, we found that the effects of testosterone on CBF measures were significantly moderated by HT use in all the regions explored (Wilks’ lambda p<0.001). In these regions, totT was positively associated with CBF in HT non-users but not in HT users (**Table 6**).

#### Associations of testosterone and cognition

Adjusting by confounders, there were no significant associations between totT and cognitive scores in men or women, before and after restricting the analysis to HT non-users (Wilks’ lambda 0.46). Focusing on regions associated with testosterone in the GMV analysis—thus restricted to men—we observed significant correlations in the left fusiform gyrus, which was positively associated with the RAVLT immediate recall test (coeff. 0.387, P = 0.008). Focusing on regions impacted by testosterone in the CBF analysis—thus restricted to women—there were no significant correlations between regional CBF and cognitive scores.

#### Whole-brain analysis

At a more liberal threshold of P<0.001, uncorrected, an additional cluster of testosterone-GMV associations was observed in the occipital lobe of the right hemisphere of the male group (**S2 Table**). In women, a positive association of totT with GMV was found in the occipital lobe of the left hemisphere, which was limited to HT non-users, whereas negative associations were observed in the sensorimotor cortex (**S2 Table**).

For CBF, in men, positive associations were found in the left middle occipital gyrus (**S3 Table**). Among all women, an additional cluster was found in the inferior occipital gyrus of the left hemisphere (**S3 Table**).

## Discussion

The present study identifies sex-specific associations of serum testosterone with GMV and CBF in AD-vulnerable regions of midlife individuals at risk of AD. Specifically, higher levels of both total and free testosterone were associated with larger GMV in men, but not in women, whereas women exhibited positive associations of testosterone with CBF, which were not observed in men. The impact of menopausal status on these associations was notably distinct, as in women, the relationship between testosterone and GMV was only evident at the premenopausal and perimenopausal stages, but not postmenopause. On the other hand, associations between testosterone and CBF were only significant from the perimenopausal stage onward. Further, testosterone-CBF associations were more pronounced among HT non-users. All results were independent of age, APOE-4 status, midlife health indicators, and SHBG levels.

Our study expands on existing literature by identifying sex-specific associations between serum testosterone levels and brain health biomarkers in midlife individuals at risk for AD. For men, our findings provide novel evidence for positive associations between both total and free testosterone and GMV, mainly in orbitofrontal cortex, temporal cortex and fusiform gyrus. The discrepancy with earlier studies on midlife men [20,21], which focused on *a priori* selected regions of interest (ROIs) and reported null findings, may be due to methodological differences. Voxel-based morphology, as utilized in our study, offers enhanced sensitivity to subtle changes while also providing subregional specificity [47], thus potentially increasing the statistical power to detect significant associations. Conversely, we did not observe significant associations between testosterone and CBF in midlife men, consistent with the only previous study in a similar population [22].

A previous study using ^15^O-labeled water positron emission tomography (PET) reported positive associations between bioavailable and total testosterone and CBF in elderly men [18], while another ^99^mTc-hexamethyl-propylene-amine-oxime (HMPAO) single-photon emission-computed tomography (SPECT) study showed increased CBF in frontal and midbrain regions of elderly hypogonadal men on testosterone therapy [50]. Therefore, it is possible that testosterone-related changes in CBF may become more pronounced at older ages. As MRI-based ASL, water-PET, and HMPAO-SPECT are all considered suitable methods for measuring CBF [51], further research is needed to compare the sensitivity of these methodologies in detecting testosterone-CBF associations in midlife men.

Findings in women displayed the opposite pattern of results than in men, with further modification effects of menopausal status and HT usage. First, we did not observe significant associations between testosterone levels and GMV across the entire female sample, in keeping with previous literature [26–28]. However, stratification by menopausal status revealed significant positive associations of testosterone with GMV in premenopausal and perimenopausal women, but not in postmenopausal women. This is in alignment with previous evidence indicating null effects in postmenopausal women [26], and larger hippocampal volume with higher testosterone levels in younger, 18–35 year old women [20]. Testosterone levels exhibit physiological fluctuations during a woman’s menstrual cycle, and correlations between circulating levels of androgens and brain androgen receptor (AR) expression have been described during the oestrus cycle of female rats [52]. Typically, women exhibit testosterone levels 10–20 times lower than men, and bioavailable testosterone levels in women roughly halve from the second to the sixth decade of life [15], a trend reflected in the hormonal assessments in our sample (premenopause 16.35 ng/dL, perimenopause 14.20 ng/dL, postmenopause 9.50 ng/dL, *p*<0.001). Together with previous null results in older postmenopausal women [26–28], this suggests that a certain amount of testosterone may be required to support neurotrophic actions [23] or produce measurable effects on brain morphology as detected with MRI. This also points to menopausal status as an important factor to consider in analysis of testosterone’s associations with brain biomarkers in midlife women. Additionally, in women, we observed positive associations between testosterone and regional CBF which were more widespread among HT non-users, encompassing both hemispheres, with peak clusters in hippocampus and orbitofrontal cortex.

Stratification by menopausal status revealed that these associations were driven by the postmenopausal and to a lesser extent perimenopausal groups, but absent at the premenopausal stage—thus displaying a somewhat opposite pattern to GMV results. Additionally, total testosterone levels were negatively associated with CBF in bilateral middle temporal gyri and right superior frontal gyrus, only at the premenopausal stage. Descriptively, in the hypothalamus, CBF was positively associated with testosterone levels in women, especially among perimenopausal women and HT users, whereas no effects were found among men. However, given the size of the hypothalamus relative to the resolution of MR-ASL, these analyses are to be considered exploratory.

The differential associations of testosterone with GMV and CBF across menopausal stages could be due to a combination of hormonal milieu, the direct versus indirect effects of testosterone, and possibly endocrine aging changes in the brain’s sensitivity to hormones. During the premenopausal and perimenopausal stages, estrogen levels are higher than in postmenopause. Both estrogen and testosterone are known to have a neurotrophic effects, promoting brain plasticity and maintaining structural integrity [2]. At these stages, testosterone’s effects on GMV may be more pronounced due to the synergistic support of estrogen or through its conversion to estrogen. In contrast, the postmenopausal stage is characterized by a decline in estrogen, while testosterone declines at a slower rate, leading to a relatively higher androgen-to-estrogen ratio [7]. In this environment, the direct effects of testosterone might become more apparent, particularly on CBF. Testosterone has been shown to influence cerebral vasculature and blood flow, potentially through vasodilation effects and modulation of vascular smooth muscle cell function [53]. Therefore, the association of testosterone with CBF postmenopause might reflect a compensatory mechanism or a shift to more direct testosterone-mediated effects due to the reduced influence of estrogen. Additionally, postmenopausal women may experience altered brain androgen and estrogen receptor sensitivities or densities, which could affect how testosterone impacts the brain. Evidence for both positive and negative associations with CBF at the premenopausal stage support this notion by suggesting presence of different hormone-mediated feedback loops in certain brain regions. This interaction might shift as hormonal levels change, disappearing in postmenopausal women where estrogen levels are lower. Interestingly, negative associations between testosterone and CBF were reported in a study of younger (30±9 year-old) women [22], and both positive and negative associations of testosterone and CBF have been found in older men [18], which warrants further investigation.

Previous studies have linked lower physiological testosterone with an increased risk of AD in both sexes [54]. In this study, the brain regions most consistently associated with testosterone levels were the orbitofrontal cortex for both men and women, the temporal cortex for men, and the hippocampus, cingulate cortex, putamen and thalamus for women. These areas are known early sites of AD pathology [46], supporting the idea that testosterone might exert a protective role against AD early on, possibly through different mechanisms in each sex. Notably, the involvement of the hippocampus—which is considered more specific to AD—among women but not men, suggests that testosterone may play a more prominent early role in modulating AD risk in midlife women. An imaging study of elderly individuals with and without cognitive impairment showed that higher testosterone levels were associated with reduced cortical Aβ positivity in women, but not in men, suggesting a greater AD-specificity of testosterone-biomarker associations in women [23]. All participants in our study have an AD family history or carry the APOE-4 genotype, both of which are established genetic risk factors for late-onset AD [29]. However, possessing genetic risk factors does not inevitably lead to the development of dementia. A natural extension of our work would be to examine biomarkers of AD pathology in relation to testosterone levels by sex, and to determine whether the observed associations between testosterone, GMV and CBF are related to or predictive of Aβ accumulation. Descriptively, at a liberal value of P<0.001, both men and women exhibited additional clusters of positive associations of testosterone with GMV and CBF in occipital cortex, and negative associations with CBF in the sensorimotor cortex among women. Both regions are typically spared by AD pathology, suggesting that associations in these regions may reflect aging rather than AD. These exploratory results deserve further investigation. For context, previous studies reported positive associations of testosterone with occipital GMV in midlife men and women [55] and in older men [17].

Another novel finding was that associations between totT and CBF were modulated by HT status (e.g., MHT or OCP usage). HT non-users exhibited stronger positive associations than HT users, whereas HT users showed generally non-significant results, except for a negative association in the right superior frontal gyrus. This is consistent with a previous study showing negative associations between testosterone and middle frontal gyrus volume in younger women using OCPs, but not in non-users [20]. HT can alter the endogenous androgen milieu, as both estrogen-containing OCPs and MHT reduce bioavailable testosterone through multiple mechanisms, primarily by decreasing androgen production by the ovaries and adrenal glands, and increasing binding to serum proteins, especially SHBG [56]. Moreover, many HT formulations include progestogens, which could interact with testosterone’s physiological effects, resulting in antiandrogenic outcomes [56]. Currently, the only FDA-approved indication for testosterone supplementation in women is for the treatment of hypoactive sexual desire disorder in postmenopause [57,58]. Only a few and relatively small randomized controlled trials (RCTs) have tested testosterone supplementation’s impact on cognition in women, providing mixed results [59]. Some of these studies included women who were also receiving estrogen therapy, which may have confounded the effects of testosterone [59]. Present results provide biomarkers for future clinical trials aimed at testing testosterone therapy to support brain function in menopausal women. They also underscore the need for further investigation into how HT interacts with endogenous testosterone, particularly within the context of aging and AD risk. Understanding the sex-dependent impacts of testosterone is valuable to developing targeted interventions that are tailored to meet the needs of precision medicine.

### Strengths and limitations

This study examines sex-specific associations of testosterone levels with brain biomarkers in midlife individuals at risk for AD, and their modulation by menopause status. We focused on carefully screened, healthy midlife men and women, aged 35–65 years, with clinical and cognitive examinations, laboratory tests, endocrine status assessments, and brain imaging. We utilized high-resolution brain scans for all participants along with state-of-the-art methods for image sampling and quantification, and conducted multivariable adjustments for several potential confounders. Our participants had an *a priori* high risk of AD, as reflected in a family history and/or APOE-4 genotype, and brain analyses focused on AD-vulnerable regions, enhancing the statistical and anatomical likelihood that the observed brain effects may be related to an AD predisposition. Future work is needed to examine whether similar associations are found among midlife individuals not at risk for AD.

Limitations of this study include its cross-sectional design, which precludes establishing causal or temporal relationships between testosterone levels and brain biomarkers. Longitudinal studies are warranted to determine whether the observed correlations are predictive of AD. As the non-interventional nature of our research prevented us from manipulating testosterone levels, clinical trials are needed to systematically explore the effects of testosterone therapy on brain biomarkers.

Due to the smaller size of the male cohort compared to the combined female cohorts, we opted not to test for sex differences in testosterone-biomarker associations. Instead, we conducted analyses within each group and described differences in association patterns qualitatively rather than quantitatively. Future research involving larger cohorts is warranted to specifically examine sex-differential effects. Additionally, we found no significant associations between testosterone levels and cognitive test scores in either group. The only previous study exploring both imaging and cognitive outcomes relative to testosterone levels was conducted in elderly men, showing associations of testosterone with GMV but not with cognitive performance [16]. However, we observed a trend toward positive associations between memory scores and GMV in fusiform gyrus of men, suggesting mediating effects of testosterone, consistent with previously observed positive effects of higher testosterone levels on cognitive performance in men [14]. No significant associations of regional CBF and cognitive performance were found in women. Nonetheless, all participants in our study had at least 12 years of education and scored within normal ranges for their age and educational level, which may have hindered detection of subtle associations between testosterone levels and cognitive scores. Furthermore, our cognitive testing battery may not have been sensitive enough to detect effects of testosterone on cognitive function at this relatively young age. Future studies with larger cohorts of participants with diverse educational and socioeconomical backgrounds, and recruited from varied community sources, are warranted to further investigate these associations.

While our analysis allowed us to differentiate by menopausal status, none of our male participants had a diagnosis of andropause, precluding stratification by andropause status. Unlike women, who inevitably undergo menopause at a mean age of 51–52, not all men experience andropause, with symptomatic androgen deficiency markedly increasing after age 70 [60]. Moreover, none of our male participants were currently undergoing testosterone therapy (TT), which precluded analysis of modulatory effects of TT on brain biomarkers. Descriptively, two men, aged 41 and 54, reported past TT use and exhibited clinical symptoms consistent with andropause, such as loss of strength, joint pain, mood changes, and decreased libido [60]. However, their testosterone values were within the normal range (totT 558 ng/dL, freeT 69 pg/mL, and totT 531 ng/dL, freeT 82 pg/mL, respectively). Excluding these two participants from analysis left results unchanged.

In this study, 21 postmenopausal women had a history of hysterectomy and/or oophorectomy, including 10 with bilateral oophorectomy, and 11 with partial hysterectomy and/or unilateral oophorectomy. Given the small sample of women with bilateral oophorectomy, we were unable to test for specific effects of surgical menopause. As oophorectomy before menopause can lead to a more abrupt decrease in testosterone levels [7], further research is needed to explore the neurophysiological impacts of such transition.

Some technical considerations also apply. As we did not acquire hematocrit (Hct) data, an assumed fixed blood T1 (T1b) of 1.6 s was used for CBF calculations. A limitation of this approach is that lower Hct levels have been associated with higher T1b [61], which could result in an overestimation of ASL-derived CBF when the assumed T1b is lower than the actual value. In healthy individuals, Hct-based T1b has been shown to be higher in women compared to men, resulting in a greater—albeit not statistically significant—difference in ASL-derived CBF measurements between sexes when using a fixed T1b [61]. Since we did not examine differences between sexes but conducted within-sex regression analyses between testosterone and CBF, any overestimation bias due to uncorrected T1b would likely act as a confounding factor rather than directly influencing the observed associations, thus conservatively reducing statistical power to detect relationships of interest. Further, our analyses were adjusted for the global mean CBF, which helps mitigate possible overestimation bias related to Hct effects on ASL-derived CBF measurements. Another factor known to elevate Hct levels is testosterone supplementation [62], which can increase blood viscosity, potentially reducing CBF in turn [62]. None of our participants reported taking testosterone therapy. Nonetheless, it is possible that higher physiological levels of testosterone may also be associated with higher Hct and, consequently, lower CBF. The absence of Hct adjustments may have thus masked positive testosterone-CBF associations in men with higher testosterone levels, which would also lead to a conservative underestimation of testosterone-CBF associations overall. None of our participants had anemia, polycythemia, or chronic kidney disease—conditions known to significantly alter Hct and, consequently, ASL measurements [61]. Further studies are needed to assess the impact of Hct adjustments on sex-specific testosterone-CBF correlations.

Our ASL scans have a reconstructed spatial resolution of 1.875x1.875x3.8 mm, which is upsampled relative to the acquisition resolution. This step, while common in clinical ASL imaging pipelines, may introduce some degree of artificial precision in the nominal resolution. However, while upsampling does not inherently improve the intrinsic spatial resolution of the data, the spatial smoothing applied in our analysis (8x8x8 mm Gaussian filter) helps to ensure that the results reflect regional perfusion patterns rather than artifacts introduced by interpolation. Additionally, we applied a cluster extent threshold of 20 voxels, which exceeds twice the effective spatial resolution of the scans, reducing the potential for false negatives. This procedure, common to voxel-based analysis [47], takes into account the application of the smoothing filter during pre-processing and is appropriate even assuming an effective spatial resolution in the order of 5x5x9 mm for ASL [63]. Moreover, significant clusters observed in our CBF analysis were generally larger than 20 voxels, with peak clusters typically exceeding 100 voxels. Finally, since we did not compare GMV and CBF results other than in a qualitative way, we did not differentiate cluster sizes between these analyses.

Considering the reconstructed spatial resolution, some partial volume contributions may occur in regions smaller than the acquired resolution. Considering our cohort’s age range of 35–65 years, presence of global atrophy is unlikely. Therefore, we opted not to perform partial volume correction on the CBF data, particularly because the significant CBF clusters differed from those identified for GMV. Specifically, in women, we observed mainly positive associations of testosterone with CBF but not with GMV. This pattern suggests that testosterone might influence blood flow independently of structural changes. As subtle GMV declines cannot be ruled out, further research is needed to examine their relative contribution to the testosterone-CBF associations, as well as the impact of regional variability in vascular contributions.

FreeSurfer was used to obtain total intracranial volumes (TIV), which were included as covariates in all GMV analyses. While tissue segmentation estimates from SPM could have been used for this purpose, we opted to use TIV derived from FreeSurfer due to its established reliability and consistency in volumetric measurements across various studies [64]. As these measures were included as covariates in the SPM design matrix rather than being directly compared or analyzed as absolute values, this approach likely helped mitigate potential biases stemming from methodological differences between the segmentation tools. We acknowledge, however, that each segmentation method has inherent advantages and limitations, and the use of different methods for obtaining TIV could introduce subtle biases. Future studies could explore the impact of these different approaches to further enhance methodological rigor.

Finally, the use of immunometric assays for measuring serum testosterone presents some limitations. These assays may overestimate testosterone levels due to interference from structurally similar steroids or nonspecific binding in the immunoassay, especially at low hormone concentrations, such as among postmenopausal women [65]. Nonetheless, this would conservatively reduce the power to detect associations between testosterone levels and brain biomarkers. Although validated methodologies were used to estimate free testosterone, it remains uncertain whether these calculations accurately reflect bioavailable levels [66]. More work is needed to examine whether alternative techniques, such as radioimmunoassay after extraction and chromatography or mass spectrometry, would yield different results.

## Conclusion

Present findings indicate sex-specific neurophysiological effects of testosterone in midlife individuals at risk for AD, with variations observed across sex and menopausal status, underscoring the need for further research focusing on the neuroprotective potential of testosterone in both sexes.

## Supporting information

S1 Fig Voxel-based statistical analysis approach for free testosterone. For free testosterone (freeT), analyses were restricted to the clusters exhibiting statistically significant associations between total testosterone (totT) and imaging outcomes in the main analysis. This was accomplished as follows: (A) We first used linear regressions to test for voxel-wise associations between totT and each imaging outcome; (B) Regional clusters showing totT effects on outcome measures were saved as binary maps (totT_MASK_) for each outcome; (C) We then used linear regressions to test for voxel-wise associations between freeT and each imaging outcome within the totT_MASK_. The example shows this procedure for the gray matter volume (GMV) outcome. Corresponding results are found in Fig 1/Table 2. Results are represented on a color-coded scale and displayed on the inferior (I), lateral left-posterior (LP), and lateral right (R) views of a volumetric MR template image with corresponding P values.
(TIF)

S2 Fig Associations between total testosterone and hypothalamic cerebral blood flow (CBF) by menopause status by premenopausal (left), perimenopausal (center), and postmenopausal (right) groups.
(TIF)

S1 Table Associations between free testosterone and regional cerebral blood flow among hormone therapy (HT) non-users. *P<0.05 cluster-level corrected for Family-Type Wise Error (FWE) within the search volume further restricted to the clusters showing significant main effects of total testosterone levels. Analyses were adjusted by age, global mean CBF, APOE-4 status, and midlife health indicators.
(DOCX)

S2 Table Whole brain associations between total testosterone and regional gray matter volume in men and women. *P<0.*001 uncorrected*. Analyses were adjusted by age, total intracranial volume, APOE-4 status, midlife health indicators and SHBG.
(DOCX)

S3 Table Whole-brain associations between total testosterone and regional cerebral blood flow in men and women. *P<0.*001 uncorrected*. Analyses were adjusted by age, global mean CBF, APOE-4 status, midlife health indicators and SHBG.
(DOCX)
