# Supplementary figures and images for: Sex-specific associations of serum testosterone with gray matter volume and cerebral blood flow in midlife individuals at risk for Alzheimer’s disease
Source: PLoS One. 2025 Jan 13;20(1):e0317303. doi: 10.1371/journal.pone.0317303 (PMC11729972; doi:10.1371/journal.pone.0317303)

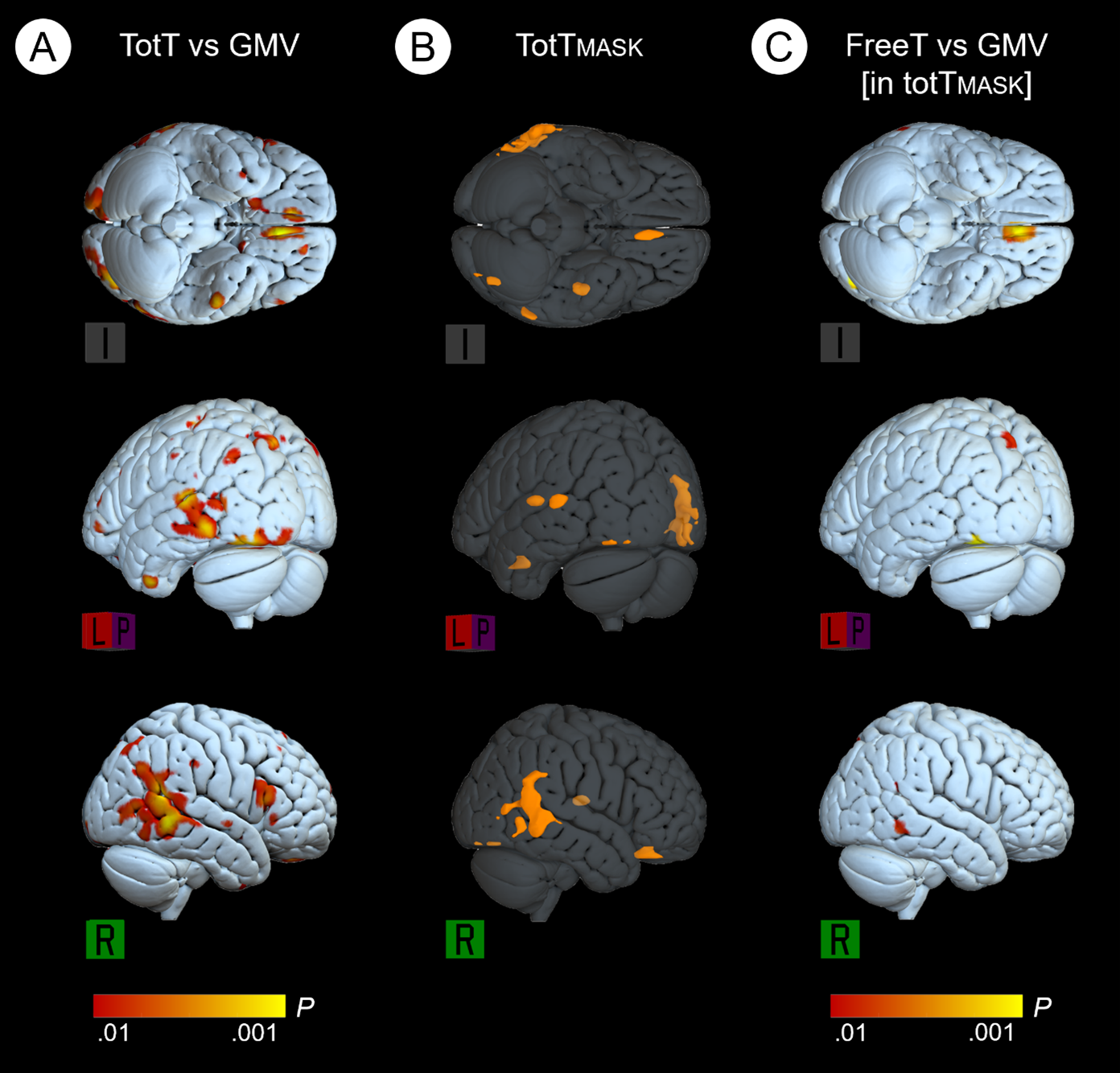

Supplement: S1 Fig — For free testosterone (freeT), analyses were restricted to the clusters exhibiting statistically significant associations between total testosterone (totT) and imaging outcomes in the main analysis. This was accomplished as follows: (A) We first used linear regressions to test for voxel-wise associations between totT and each imaging outcome; (B) Regional clusters showing totT effects on outcome measures were saved as binary maps (totTMASK) for each outcome; (C) We then used linear regressions to test for voxel-wise associations between freeT and each imaging outcome within the totTMASK. The example shows this procedure for the gray matter volume (GMV) outcome. Corresponding results are found in Fig 1/Table 2. Results are represented on a color-coded scale and displayed on the inferior (I), lateral left-posterior (LP), and lateral right (R) views of a volumetric MR template image with corresponding P values. (TIF) [file pone.0317303.s001.tif]

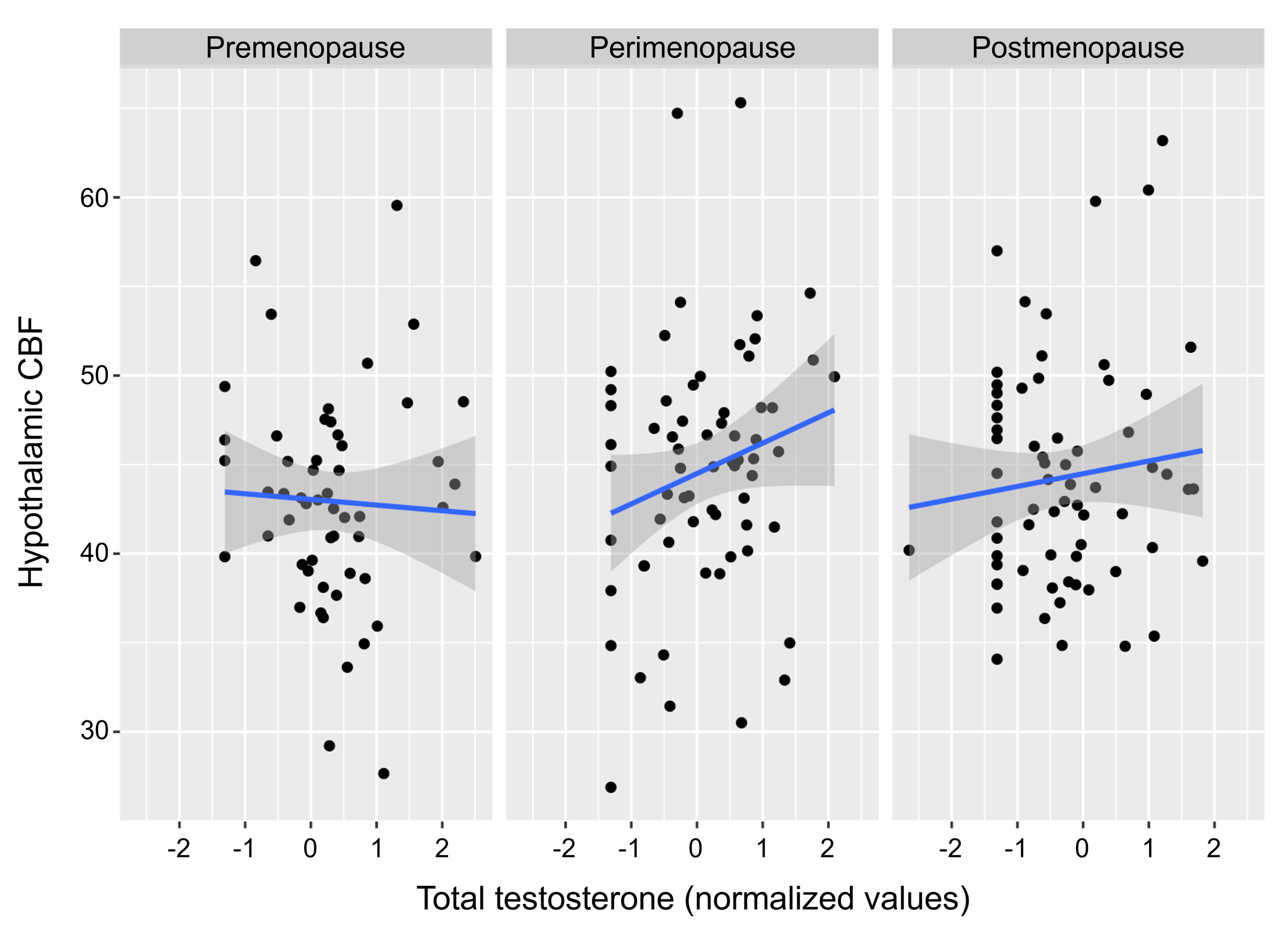

Supplement: S2 Fig — Associations between total testosterone and hypothalamic cerebral blood flow (CBF) by menopause status by premenopausal (left), perimenopausal (center), and postmenopausal (right) groups. (TIF) [file pone.0317303.s002.tif]
