# Supplementary material for: Sex-specific associations of serum testosterone with gray matter volume and cerebral blood flow in midlife individuals at risk for Alzheimer’s disease
Source: PLoS One. 2025 Jan 13;20(1):e0317303. doi: 10.1371/journal.pone.0317303 (PMC11729972; doi:10.1371/journal.pone.0317303)
Supplement: S1 Table — *P<0.05 cluster-level corrected for Family-Type Wise Error (FWE) within the search volume further restricted to the clusters showing significant main effects of total testosterone levels. Analyses were adjusted by age, global mean CBF, APOE-4 status, and midlife health indicators. (DOCX) [file pone.0317303.s003.docx]

**S1 Table. Associations between free testosterone and regional cerebral blood flow among hormone therapy (HT) non-users.**

| Cluster extent | Coordinates x, y, z | Z | P_FWE_ cluster* | P voxel | Anatomical Region |
| --- | --- | --- | --- | --- | --- |
| **Positive associations** | | | | | |
| 107 | -21 -36 -8 | 4.07 | 0.006 | <0.001 | Hippocampus, left |
|  | -15 -36 -2 | 3.45 |  | <0.001 | Hippocampus, left |
| 16 | 26 54 -14 | 3.90 | 0.018 | <0.001 | Orbitofrontal cortex, right |
| 136 | 12 14 -9 | 3.57 | 0.005 | <0.001 | Putamen, right |
| **Negative associations** | | | | | |
| n.s. | | | | | |

**P<0.05* cluster-level corrected for Family-Type Wise Error (FWE) within the search volume further restricted to the clusters showing significant main effects of total testosterone levels. Analyses are adjusted by age, global mean CBF, APOE-4 status, and midlife health indicators.
