# Supplementary material for: Sex-specific associations of serum testosterone with gray matter volume and cerebral blood flow in midlife individuals at risk for Alzheimer’s disease
Source: PLoS One. 2025 Jan 13;20(1):e0317303. doi: 10.1371/journal.pone.0317303 (PMC11729972; doi:10.1371/journal.pone.0317303)
Supplement: S2 Table — *P<0.001 uncorrected. Analyses were adjusted by age, total intracranial volume, APOE-4 status, midlife health indicators and SHBG. (DOCX) [file pone.0317303.s004.docx]

**S2 Table. Whole brain associations between total testosterone and regional gray matter volume in men and women**

| Cluster extent | Coordinates x, y, z | Z | Anatomical Region |
| --- | --- | --- | --- |
| **Men** | | | |
| ***Positive associations*** | | | |
| 210 | -6 33 -27 | 3.98 | Orbitofrontal cortex, left |
|  | -6 44 -33 | 3.66 | Orbitofrontal cortex, left |
| 140 | -40 -87 -16 | 3.91 | Fusiform gyrus, left |
|  | -39 -76 -18 | 3.17 | Fusiform gyrus, left |
| 31 | -48 6 46 | 3.78 | Middle frontal gyrus, left |
| 30 | 63 4 33 | 3.73 | Inferior frontal gyrus, right |
| 36 | -64 -54 10 | 3.54 | Middle temporal gyrus, left |
| 28 | 8 42 -27 | 3.53 | Orbitofrontal cortex, right |
| 122 | 8 -98 -2 | 3.53 | Lingual gyrus, right |
| 40 | -44 -15 14 | 3.49 | Inferior frontal gyrus, left |
| 39 | 69 -45 -3 | 3.46 | Middle temporal gyrus, right |
| 38 | 64 -51 18 | 3.40 | Superior temporal gyrus, right |
| ***Negative associations*** | | | |
| n.s. | | | |
| **Entire cohort of women** | | | |
| ***Positive associations*** | | | |
| n.s. | | | |
| ***Negative associations*** | | | |
| 55 | 22 -34 68 | 3.92 | Postcentral gyrus, right |
| 25 | -33 -44 57 | 3.67 | Postcentral gyrus, left |
| 22 | -12 27 40 | 3.45 | Superior frontal gyrus, left |
| **Women HT non-users** | | | |
| ***Positive associations*** | | | |
| 27 | 3 -76 24 | 3.60 | Cuneus, left |
| ***Negative associations*** | | | |
| 123 | -45 -2 44 | 4.46 | Precentral gyrus, left |
| 33 | -33 -44 58 | 3.92 | Postcentral gyrus, left |
| 34 | 24 -15 64 | 3.64 | Precentral gyrus, right |
| 30 | 30 -32 63 | 3.33 | Postcentral gyrus, right |

**P<0.001 uncorrected.* Analyses are adjusted by age, total intracranial volume, APOE-4 status, midlife health indicators and SHBG.
