# Supplementary material for: Sex-specific associations of serum testosterone with gray matter volume and cerebral blood flow in midlife individuals at risk for Alzheimer’s disease
Source: PLoS One. 2025 Jan 13;20(1):e0317303. doi: 10.1371/journal.pone.0317303 (PMC11729972; doi:10.1371/journal.pone.0317303)
Supplement: S3 Table — *P<0.001 uncorrected. Analyses were adjusted by age, global mean CBF, APOE-4 status, midlife health indicators and SHBG. (DOCX) [file pone.0317303.s005.docx]

**S3 Table. Whole-brain associations between total testosterone and regional cerebral blood flow in men and women.**

| Cluster extent | Coordinates x, y, z | Z* | Anatomical Region |
| --- | --- | --- | --- |
| **Men** | | | |
| ***Positive associations*** | | | |
| 28 | -30 -87 33 | 3.31 | Middle occipital gyrus, left |
| ***Negative associations*** | | | |
| n.s. | | | |
| **Entire cohort of women** | | | |
| ***Positive associations*** | | | |
| 112 | -18 -36 -4 | 3.67 | Hippocampus, left |
| 100 | -48 -70 -18 | 3.58 | Inferior occipital gyrus, left |
| 107 | -42 -28 8 | 3.48 | Superior temporal gyrus, left |
|  | -38 -33 20 | 3.23 | Superior temporal gyrus, left |
| ***Negative associations*** | | | |
| 274 | 54 34 16 | 4.11 | Inferior frontal gyrus, right |
|  | 51 46 9 | 3.43 | Middle frontal gyrus, right |
| 110 | -16 21 14 | 3.66 | Caudate, left |
| **Women HT non-users** | | | |
| ***Positive associations*** | | | |
| 258 | -21 -36 -4 | 4.50 | Hippocampus, left |
| 491 | 12 14 -10 | 4.01 | Inferior frontal gyrus, right |
|  | 16 15 -18 | 3.71 | Orbitofrontal cortex, right |
|  | 16 6 6 | 3.15 | Putamen, right |
| 370 | -3 -16 0 | 3.90 | Thalamus, left |
|  | -3 -16 -12 | 3.87 | Thalamus, left |
| ***Negative associations*** | | | |
| 268 | 54 36 16 | 3.69 | Inferior frontal gyrus, right |
|  | 52 45 4 | 3.46 | Middle frontal gyrus, right |

**P<0.001 uncorrected.* Analyses are adjusted by age, global mean CBF, APOE-4 status, midlife health indicators and SHBG.
